# Novel Role of Molecular Hydrogen: The End of Ophthalmic Diseases?

**DOI:** 10.3390/ph16111567

**Published:** 2023-11-07

**Authors:** Si-Yu Li, Rong-Yue Xue, Hao Wu, Ning Pu, Dong Wei, Na Zhao, Zong-Ming Song, Ye Tao

**Affiliations:** Henan Eye Institute, Henan Eye Hospital, People’s Hospital of Zhengzhou University, Henan Provincial People’s Hospital, Zhengzhou 450003, China

**Keywords:** molecular hydrogen, ophthalmic diseases, oxidation stress

## Abstract

Molecular hydrogen (H_2_) is a colorless, odorless, and tasteless gas which displays non-toxic features at high concentrations. H_2_ can alleviate oxidative damage, reduce inflammatory reactions and inhibit apoptosis cascades, thereby inducing protective and repairing effects on cells. H_2_ can be transported into the body in the form of H_2_ gas, hydrogen-rich water (HRW), hydrogen-rich saline (HRS) or H_2_ produced by intestinal bacteria. Accumulating evidence suggest that H_2_ is protective against multiple ophthalmic diseases, including cataracts, dry eye disease, diabetic retinopathy (DR) and other fields. In particular, H_2_ has been tested in the treatment of dry eye disease and corneal endothelial injury in clinical practice. This medical gas has brought hope to patients suffering from blindness. Although H_2_ has demonstrated promising therapeutic potentials and broad application prospects, further large-scale studies involving more patients are still needed to determine its optimal application mode and dosage. In this paper, we have reviewed the basic characteristics of H_2_, and its therapeutic effects in ophthalmic diseases. We also focus on the latest progress in the administration approaches and mechanisms underlying these benefits.

## 1. Introduction

Molecular hydrogen (H_2_) is a colorless, odorless and tasteless diatomic gas with small molecular size. H_2_ can readily cross the biologic barriers, penetrate the tissue and efficiently remove surplus reactive oxygen species (ROS) [1,2]. In addition to its antioxidative capacity, H_2_ also has the characteristics of anti-inflammation, anti-apoptosis and cytoprotection in mitochondria [3]. Emerging animal experiments and clinical studies have utilized the H_2_ in the treatment of ocular diseases and have demonstrated promising therapeutic potential. For instance, H_2_ can prevent a reduction in tear stability in patients and exerts therapeutic effects on the oxidative ocular diseases in animal models.

ROS are produced via the partial reduction of molecular oxygen under normal physiological conditions [4]. ROS encompass molecules derived from O_2_, including superoxide (O_2_^•−^), hydrogen peroxide (H_2_O_2_), hydroxyl radical (•OH), ozone and singlet oxygen [5]. Typically, oxidative stress is caused by excessive ROS production, mitochondrial dysfunction, an impaired antioxidant system or a combination of these factors [6,7,8]. Excessive ROS can disrupt the redox homeostasis and result in the production of a large amounts of peroxynitrite (ONOO^−^) and •OH [9]. Peroxynitrite acid (ONOOH) is a form of protonation of ONOO^−^, a strong oxidant that can exacerbate the risk of oxidative stress in the body [9]. At the same time, ONOOH can also be decomposed to form intermediates (•OH and •NO_2_) [9]. Both •NO_2_ and •OH are indiscriminate in what they will oxidize, which creates the havoc called oxidative stress [5]. In this context, excessive ROS will cause oxidative damage to deoxyribonucleic acid (DNA), proteins and lipids. For instance, ROS can react with the nitrogen-containing bases of nucleic acids and the glycosylphosphate skeleton of DNA, resulting in chromosome and mitochondrial DNA (mtDNA) damage [10]. DNA damage can affect the protein-coding region and non-coding regulatory region of the gene, thus affecting protein expression and regulation [11,12]. DNA damage can also activate the IκB kinase (IKK) complex, resulting in the phosphorylation, ubiquitination and degradation of IκBα and the activation of nuclear factor kappaB (NF-kB) [13]. Oxidative stress produces reactive oxygen intermediates (ROI), which activate caspase-1, causing the proteolytic cleavage of NF-kB proteins, leading to apoptosis [14]. mtDNA damage causes further respiratory chain dysfunction and increase membrane permeability, resulting in the excessive production of ROS and a continuous oxidative stress cycle [15]. ROS also attack the structural and enzymatic proteins by oxidizing residual amino acids and pseudogroups, thereby forming cross-links, protein aggregates and proteolysis [16]. The inactivation of these proteins can lead to serious abnormalities in key metabolic pathways. Lipid peroxidation is the oxidation process of polyunsaturated fatty acids (PUFAs) [16,17]. Due to the existence of multiple double bonds in PUFA structures, lipid peroxidation is involved in the production of peroxides (compounds in which two oxygen atoms are connected via a single covalent bond), ROS and other active organic free radicals. Lipid peroxidation can also induce apoptosis, ferroptosis and autophagy during the cell death [18].

In the past few decades, oxidative stress has been recognized as a critical pathogenic factor of various ocular diseases, such as the endothelial corneal dystrophy, terygium, glaucoma, cataracts, uveitis, retinopathy and optic neuropathies [4,19]. Oxidative stress can impair tissue structure, increase vascular permeability and promote neovascularization in the eye [20,21,22,23]. Moreover, oxidative stress can initiate inflammatory response, ocular cell apoptosis and ferroptotic cell death [14,24,25]. In this context, oxidative stress may act as an effective target for the treatment of ocular disease.

## 2. Advantages and Potentials of Molecular Hydrogen in Treating Ocular Diseases

**Figure 1:**
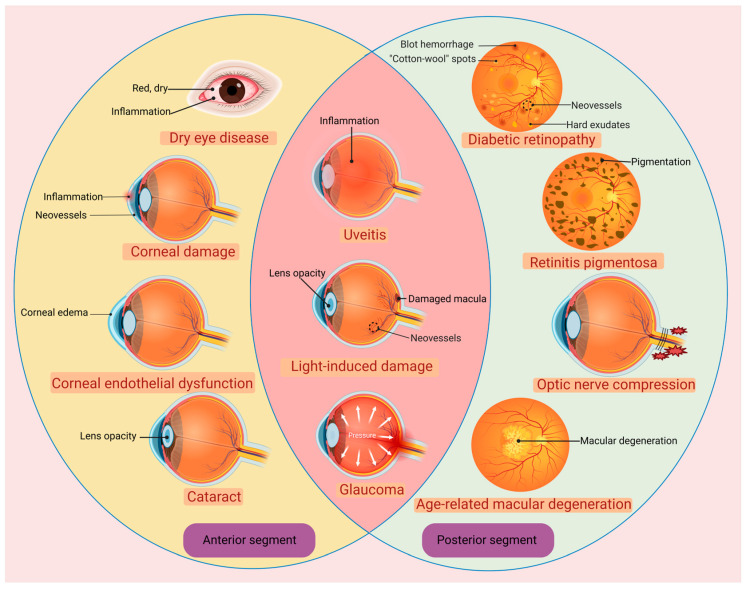
The emerging applications of hydrogen in the treatment of various ophthalmological diseases. Created with BioRender.com.

The molecular weight of H_2_ is 2.01588, which is the smallest in nature. The density of H_2_ is 0.089 g/L (101.325 kpa, 0 °C), which is 1/14 of the same volume of air [26]. The small molecular weight and low density endow H_2_ with an extremely strong ability to penetrate tissue rapidly. H_2_ behaves as an inert gas in the presence of catalysts at body temperature [1]. Only strong oxidants, like ONOO^−^, are able to oxidize H_2_ [3]. On these bases, H_2_ is very mild and safe in human tissue, neither disturbing normal metabolic redox reactions nor affecting the physiological function of ROS in cell signaling [3]. Once excessive ROS are produced to induce oxidative stress in cells, H_2_ can efficiently attack •NO_2_ and •OH, which are the main cytotoxic ROS [27]. In 2007, Ohsawa et al. reported for the first time that H_2_ is a selective antioxidant when administered via gaseous rapid diffusion into tissues and cells [2]. Unlike other therapeutic gases, such as NO, CO or hydrogen sulfide, H_2_ will not induce any toxicity even at high concentrations [1]. These advantages make H_2_ an excellent candidate for treating ocular diseases (Figure 1). In ophthalmological practice, the dynamic and static biologic barriers are the most challenging, since they will affect the bioavailability of drugs in both the anterior and posterior segments. These biologic barriers will retract the passive absorption and reduce the delivery efficiency of therapeutic agents [28]. For instance, eye drops account for about 90% of ophthalmic medications for anterior segment eye diseases, but only 1–5% of the applied drug remains on the ocular surface for a sufficiently long time [29]. Furthermore, the static and dynamic barriers of the eye hinder the penetration of various therapeutic agents, including conventional small molecules and macromolecules, such as antioxidants (e.g., superoxide dismutase, catalase and vitamin C); anti-inflammatory agents (e.g., cefazolin, amikacin); anti-aggregation agents, like protein chaperones (e.g., clusterin and crystallins); small molecule chaperones (e.g., quinacrines, curcumin and chlorpromazine), etc. [30,31].

The static barrier of the cornea is the outermost part of the ocular barrier. It is composed of the lipophilic corneal epithelium, hydrophilic corneal stroma, corneal endothelium and blood aqueous barrier [28]. Although the lipophilic corneal epithelium allows the penetration and absorption of hydrophobic drugs, the paracellular diffusion will still be restricted by the tight junctions between corneal epithelium cells [32,33]. Simultaneously, some efflux transporters in the plasma membrane of corneal epithelial cells also reduce the transportation rate of drugs [34]. Moreover, the dynamic barriers such as tear drainage, conjunctival lymph, blood and aqueous humor circulation would reduce sharply the penetrate efficiency of therapeutic molecules [29]. Even if a small proportion of drugs bypass the corneal barrier, the conjunctiva, sclera, choroid, Bruch’s membrane and retinal barrier in the anatomic pathway are other conundrums [29]. In particular, the thickness of Bruch’s membrane can increase with age, showing a gradually stronger blocking ability [28]. The blood–retinal barrier (BRB) is another challenge for efficient drug delivery [35]. BRB consists of the internal and external barriers. The internal barrier is composed of the adherens junction and zonula occludens between vascular endothelial cells. The external barrier is composed of the RPE cells and their connections, which can separate choroid interstitial fluid from retinal interstitial fluid [28]. Collectively, these layered barriers hinder the transportation of most therapeutic drugs into the eye. However, H_2_ can quickly cross these biologic barriers and enter cellular membranes and even mitochondria and nuclei due to its powerful penetrating ability [1]. Additionally, H_2_ can scavenge the ROS selectively on the basis of its unique reducibility in a selective manner without cytotoxicity. Hence, owing to its superior characteristics, H_2_ gas holds immense potential for extensive investigation into diverse ophthalmic diseases (Table 1).

## 3. Mechanisms Underlying the Therapeutic Effects of Molecular Hydrogen

**Figure 2:**
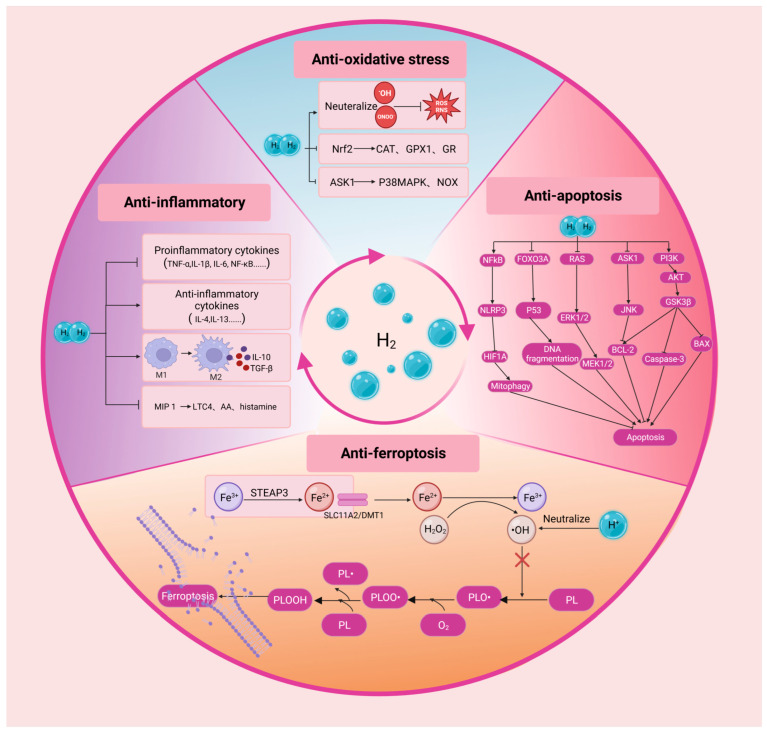
The fundamental principles underlying the therapeutic effects of H_2_ in ophthalmological pathologies. H_2_, molecular hydrogen; •OH, hydroxyl radical; ONOO^−^, peroxynitrite; ROS, reactive oxygen species; Nrf2, nuclear factor erythroid 2-related factor 2; CAT, catalase; GPX1, glutathione peroxidase 1; GR, glutathione reductase; ASK1, apoptosis signal-regulating kinase 1; P38MAPK, p38 mitogen activated protein kinase; NOX, NADPH Oxidases; TNF-α, tumor necrosis factor alpha; IL, interleukin; NF-κB, nuclear factor kappaB; TGF-β, transforming growth factor-beta; MIP 1, macrophage inflammatory protein 1.LTC4, leukotriene C4; AA, arachidonic acid; STEAP3, six-transmembrane epithelial antigen of the prostate 3; SLC11A2, solute carrier family 11 member 2; DMT1, divalent metal transporter 1; H_2_O_2_, hydrogen peroxide; PL, phospholipid; PLOOH, phospholipid hydroperoxides; NLRP3, NOD-like receptor family pyrin domain containing 3; RAS, rat sarcoma; ERK1/2, extracellular signal-related kinases 1 and 2; JNK, Jun N-terminal kinase; BCL-2, B cell lymphoma protein-2; PI3K, phosphatidylinositol 3-kinases; GSK3β, glycogen synthase kinase-3beta. Created with BioRender.com.

H_2_ can readily penetrate biofilms compared to classic anti-oxidants, such as superoxide dismutase (SOD), catalase (CAT) and alpha-tocopherol, and H_2_ is able to specifically eliminate cytotoxic free radicals (Figure 2) [58]. In order to determine the free radical species reduced directly by H_2_, researchers have tested the reactivity of H_2_ with artificially prepared ROS or reactive nitrogen species (RNS) in cell-free experiments. The results show that H_2_ can directly neutralize •OH and generate water, thereby inhibiting the primary free radical chain reactions. However, H_2_ does not affect directly the levels of O_2_^•−^, H_2_O_2_ and •NO, all of which play essential roles in signal transduction [2]. In addition to directly alleviating oxidative stress, H_2_ can also promote the expression of endogenous antioxidant enzymes. Nuclear factor erythroid 2-related factor 2 (Nrf2) is a basic region leucine-zipper transcription factor that plays a pivotal role in the coordinated gene expression of antioxidant enzymes [59]. H_2_ can promote Nrf2 transcriptional activation and induce its translocation into the nucleus, thereby enhancing the transcription of anti-oxidative enzymes such as CAT, glutathione 1 and glutathione reductase [26]. Furthermore, H_2_ participates in the metabolic processes related to oxidation–reduction reactions. H_2_ can decrease the levels of NADPH oxidase subunits, including p40 phox, p47 phox and p67 phox in the cell membrane, but increase their levels in the cytoplasm. By limiting the translocation of these molecules to the cell membrane, H_2_ reduces the NADPH oxidase activity [26]. H_2_ can also inhibit the activity of NADPH oxidase by blocking the apoptosis signal-regulating kinase 1 (ASK1) pathway and the downstream mediator p38 mitogen-activated protein kinase [60]. Thus, H_2_ can indirectly decrease the production of ROS by inhibiting NADPH oxidase activity.

H_2_ also exerts protective effects through anti-inflammatory properties. Emerging evidence suggests that H_2_ can inhibit the release of proinflammatory cytokines, including interleukin (IL)-1β, IL-6, prostaglandin E_2_, tumor necrosis factor-α (TNF-α), NF-κB, interferon-γ and high-mobility group box 1 [3,26,61]. It can also enhance the expression level of anti-inflammatory cytokines, such as IL-4 and IL-13 [26,61]. Moreover, H_2_ facilitates macrophage polarization from proinflammatory M1 type to anti-inflammatory M2 type, which in turn generates additional anti-inflammatory cytokines, including IL-10 and transforming growth factor-β [62]. A recent study show that H_2_ is able to enhance macrophage-mediated phagocytosis and inhibit the neutrophil recruitment at lesion location s [60]. Moreover, H_2_ has been documented to alleviate the inflammation by mitigating the expression level of macrophage inflammatory protein 1 (MIP 1) family members [61]. MIP 1 exacerbates the acute and chronic inflammatory responses when the tissue is exposed to trauma or infection. This cytokine can induce the release of proinflammatory mediators such as leukotriene C4, arachidonic acid or histamine [63].

H_2_ can inhibit the activation of apoptotic cascades in multiple types of tissue. Apoptosis is caused by the successive activation of a series of cysteine proteases known as caspases [60]. Apoptosis is involved in the pathology of various ophthalmological disease, such as glaucoma, retinitis pigmentosa (RP), cataracts, retinoblastoma, retinal ischemia and diabetic retinopathy (DR) [64]. H_2_ can protect ocular tissue by modulating the expression of apoptosis-related factors, such as inhibiting the expressions of proapoptotic factors BAX, caspase-3, caspase-8 and caspase-12, while upregulating the expressions of antiapoptotic factors, such as B cell lymphoma protein-2 (BCL-2) and B-Cell lymphoma-extra-large (BCL-XL) [26]. H_2_ also inhibits apoptosis by the regulating signal transduction within and between specific pathways. For example, the H_2_-mediated neuroprotective effect is at least partially associated with anti-apoptotic protein kinase B (AKT) pathway activation in neurons [3]. In addition, there are other signaling pathways that play a critical role in H_2_ inhibiting cell apoptosis, such as PI3K/Akt/GSK3β, ASK1/JNK and Ras-ERK1/2-MEK1/2 pathways. Notably, H_2_ can promote apoptosis in some cases. Apoptosis evasion is a prominent hallmark of cancer, where H_2_ inhibits tumor cell proliferation and migration as well as invasion and promotes cell apoptosis in lung cancer [65].

Ferroptosis is a cell death modality that is characterized by the iron-dependent accumulation of lipid peroxidation. Ferroptosis is closely related to the pathogenesis of various ocular diseases. For example, the progressive death of RPE cells and overlying photoreceptors is the hallmark feature of age-related macular degeneration (AMD) pathology [66]. Recent studies suggest that ferroptosis is the momentous mechanism underlying the RPE/photoreceptor cells death in AMD [67]. Malondialdehyde (MDA) is one of the final products of polyunsaturated fatty acids peroxidation. A study shows that drinking hydrogen-rich saline (HRS) can significantly reduce the serum MDA level of older adults [68]. 4-hydroxynonenal (4-HNE) is the most well-studied and seemingly the most relevant product of lipid peroxidation in ferroptosis [69]. Encouragingly, researchers find that H_2_ dissolved in medium can decrease the levels of 4-HNE and alleviating the pheochromocytoma cells death in a dose-dependent manner [2]. In addition to protecting cell integrity by reducing the production of lipid peroxidation elements, H_2_ can also inhibit ferroptosis by ameliorating oxidative stress. This should be ascribed to some mutual commonalities and overlap between the H_2_ signaling and ferroptosis pathways [70]. Oxidative stress can induce the lipid peroxidation of phospholipid (PL) in the cell membrane, leading to the ferroptosis of cells. H_2_ may significantly block the occurrence of this process by inhibiting lipid peroxidation [70]. In this context, the anti-ferroptosis mechanism should be partially responsible for the H_2_-mediated beneficial effects.

## 4. Current Administration Approaches of Molecular Hydrogen

### 4.1. Hydrogen Gas

**Figure 3:**
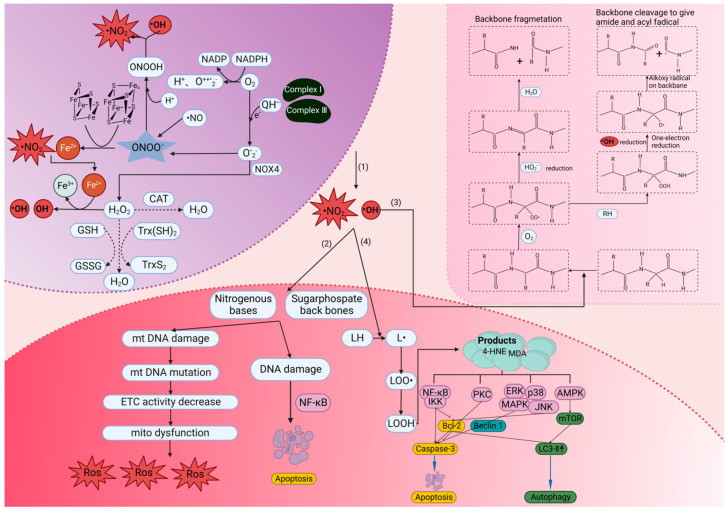
The mechanism of oxidative stress and its damage to DNA, lipids and proteins. (1) •OH and ONOO^−^ production mechanism. (2) Damage of chromosome and mitochondrial DNA by •OH and •NO_2_ and its possible consequences. (3) The α-carbon site of amino acid residues can form stable carbon-centered radicals with hydroxyl radicals and react with O_2_ to form alkyl peroxyl groups. Peroxyl radicals can be removed via an elimination reaction that releases HO_2_• and generates an imine, which subsequently undergoes hydrolysis and thus gives rise to backbone fragmentation. In addition, peroxyl radicals can extract hydrogen atoms from another species to produce hydroperoxide. The subsequent decomposition of these hydroperoxides to radicals can also result in backbone fragmentation via an alkoxyl–radical-mediated process. (4) LH is a lipid with allylic hydrogens, which are present in polyunsaturated fatty acids, including arachidonic acid. The reaction of •OH and LH leads to lipid peroxidation and eventually causes autophagy and apoptosis. LH, luteinizing hormone; CAT, catalase; L•, an alkoxyl radical; LOO•, peroxyl radical; LOOH, a lipid hydroperoxide; QH•−, ubisemiquinone; •OH, hydroxyl radical; H_2_O_2_, hydrogen peroxide; O_2_^•−^, superoxide; ONOO^−^, peroxynitrite; •NO, nitric oxide; HO_2_•, hydroperoxyl radical; ETC, electron transport chain; NOX4, NADPH oxidase 4; ONOOH, peroxynitrous acid; MAPKs, mitogen-activated protein kinases; JNK, Jun N-terminal kinase; ERK, extracellular signal-regulated kinase; PKC, protein kinase C; AMPK, adenosine monophosphate-activated protein kinase; mTOR, mammalian target of rapamycin; IKK, IκB kinase; NF-κb, nuclear factor κB. Created with BioRender.com.

In the pioneering studies, H_2_ gas can be produced through water electrolysis. Currently, the common approaches of H_2_ gas therapy include delivery through a facemask, nasal cannula or ventilator circuit [71,72,73]. H_2_ gas therapy has demonstrated many advantages in the treatment of certain diseases. Firstly, it acts rapidly and is particularly suitable for addressing acute oxidative stress. ROS can cause damage to cellular components (Figure 3). The main advantage of H_2_ gas relies on its ability to penetrate rapidly cell membranes and enter the organelles, where it specifically targeting and neutralizing cytotoxic ROS [2,74]. Secondly, H_2_ gas therapy does not have adverse effects on blood pressure, which is recognized as a crucial risk factor under certain conditions. H_2_ gas therapy does not produce side effects on the cardiovascular system, making it a relatively safe treatment option [2]. However, H_2_ gas therapy also has some drawbacks. Firstly, it is not as convenient as other portable medications or treatment devices. H_2_ needs to be prepared and stored using gas generators or pressure tanks, which makes H_2_ gas therapy challenging to implement in certain situations. Secondly, there is potential risk of explosion when using H_2_ gas. Although H_2_ gas leakage is rare in clinical practice, the careful management of the delivery process is necessary to ensure the safety of patients and healthcare professionals. Thus, the definitive quantitative data and uniform standards for the application of H_2_ gas in ophthalmic practice remain unset. Typically, scientific studies involving the inhalation of H_2_ gas require diluting the H_2_ gas to an appropriate ratio, with 66% H_2_ gas being used in some ophthalmology animal experiments [42,49]. However, the precision in defining both the volume and intensity of H_2_ inhalation, under secure conditions, and its further applications in the treatment of ophthalmic diseases, as opposed to other ailments, remain a domain that requires exploration and experimentation. In this context, H_2_ gas administration has some limitations which might impede its application and therapeutic efficacy in clinical practice.

### 4.2. Hydrogen-Rich Water/Hydrogen-Rich Saline

Hydrogen-rich water (HRW)/HRS refers to water or saline with a higher concentration of H_2_. Currently, several common methods have been developed to produce HRW/HRS. One method is to inject H_2_ into water or saline (usually 0.9% NaCl solution) under high pressure, allowing the water or saline to dissolve the H_2_. Another method is to produce H_2_ through electrolysis and then dissolve it in water or saline. The third method mainly involves the reaction of metal elements with acids (primarily sulfuric acid and hydrochloric acid). By utilizing metals with higher reactivity than H_2_, atoms are displaced from H_2_O to generate H_2_ gas. Finally, these preparation procedures enrich the water or saline with H_2_ and endow it with therapeutic potentials. The quantity of H_2_ dissolved in HRW or HRS can vary according to the preparing method. Typically, HRW/HRS used in experiments contains a dissolved H_2_ concentration of 0.6 mM (1.2 ppm) or above [44,47].

**Figure 4:**
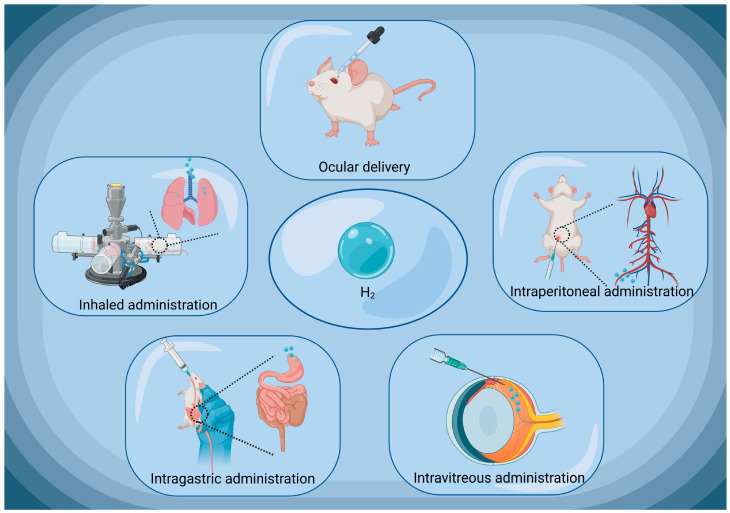
Common routes of H_2_ administration in ophthalmology. H_2_, molecular hydrogen. Created with BioRender.com.

HRW/HRS can be administered in various ways, such as through oral intake, drops, or direct injection [38,75,76] (Figure 4). In ophthalmologic practice, HRW/HRS is always applied through local eye drops. Through this delivery approach, HRW/HRS can directly contact the ocular tissues, rapidly penetrate corneal epithelium and enter the interior eyeball [36]. Experimental results have shown that when HRS eye drops are typically administered on the ocular surface of rat pups, a certain amount of H_2_ can be detected in the vitreous humor. Only two minutes after administration, the concentration of H_2_ in the vitreous humor begins to increase and peaks at 15 min. At this point, the H_2_ concentration in the vitreous humor is approximately 20% of that in the eye drops [77]. Additionally, HRW/HRS can also be administered intravitreal (IV) injections [56]. Unlike oral intake, in which H_2_ can both escape over time and be lost in the stomach or intestines, IV injections achieve a more precise control of the H_2_ concentration [78]. The precise locale for the injection on the ocular surface is determined, typically about 4 mm from the limbus. Once the needle has secured its position properly, the HRS is judiciously released into the vitreous humor. In this approach, HRW/HRS is directly injected into the vitreous humor of the eye, placing it in direct contact with intraocular cells. This administrative method is typically used for posterior segment pathologies, which is challenging for conventional delivery approach.

HRW/HRS is advantageous in the treatment of ocular diseases. Firstly, it is portable, convenient and safe. Patients can carry HRW/HRS with them without the need for complex equipment or special administrative procedures [79]. Secondly, the therapeutic effect of HRW/HRS is comparable to that of inhaling H_2_ gas. H_2_ affords antioxidant effects by selectively neutralizing the toxic free radicals in the body. Through oral intake or direct contact with HRW/HRS, the body can absorb water enriched with H_2_, thereby achieving the comparable effects of inhaling H_2_ gas [80]. Notably, HRW/HRS administration also has some drawbacks: firstly, H_2_ gradually escapes form the water over time, making it difficult to store HRW/HRS for an extended period of time. Therefore, patients need to consume or use HRW/HRS within a certain time frame to ensure its efficacy. Secondly, when taken orally, some H_2_ may be lost, making it challenging to precisely control the H_2_ concentration. Compared to inhaling H_2_ gas directly, the oral intake of HRW/HRS may result in reduced absorption efficiency. Furthermore, although HRW/HRS can be administered more easily, the duration of H_2_ retention in the body is only approximately 20–30 min after administration, which cannot provide a reliable supply of H_2_ like the direct inhalation of H_2_ gas [81]. Therefore, careful dosage and frequency control are necessary during HRW/HRS treatment. In clinical practice, a comprehensive consideration of its strengths and limitations is required to ensure that patients obtain an effective concentration of H_2_.

### 4.3. Molecular Hydrogen Produced by Intestinal Bacteria

The direct use of H_2_ gas poses some safety risks and inconveniences. On the other hand, the short duration of HRW in the body limits its application in clinical practice (Table 2) [81]. In recent years, a novel delivery method has gained abundant interest, in which the researcher utilizes gut bacteria to produce a large amount of H_2_ in the body. When indigestible components reach the colon, gut bacteria utilize them to produce H_2_, carbon dioxide, short-chain fatty acids, and other endogenous substances [82]. Previous studies have shown that H_2_ produced by gut microbiota can influence the central nervous system [83]. Experimental evidence has suggested that certain bifidobacteria in mouse intestines may affect H_2_ production through the ingestion of dietary substances [84].

This method provides new possibilities for the convenient application of H_2_ in the treatment of ophthalmic diseases. Researchers have used the H_2_-producing milk to prevent the short-fluorescein tear film breakup time (fTBUT) type of dry eye symptom, providing new insights into the clinical application of H_2_ [85]. Previous studies have shown that pectin and high amylose corn starch can increase H_2_ production in the cecum of rats [86]. Moreover, the results of a clinical trial also show that eating turmeric can also increase the production of respiratory H_2_ [87]. It is reasonable to speculate that these substances may also be used like H_2_-rich milk in the future.

There are more than 100 trillion kinds of intestinal bacteria from 1000 different species in our large intestine. Among these bacteria, 70% are H_2_-producing bacteria [88]. They can ferment substances that cannot be absorbed in the intestines into short-chain fatty acids, releasing carbon dioxide and H_2_. In the colon, three different types of microorganisms, including methane-producing bacteria, sulfate-reducing bacteria and acetate-forming bacteria can consume the H_2_ in the gut [89,90,91]. Under physiologic conditions, they do not receive enough H_2_, which hinders their significant proliferation. However, once the production of H_2_ in the gut is enhanced, the H_2_-consuming bacteria will multiply accordingly, resulting in a decrease in the H_2_ concentration expelled from the colon [92]. Although certain bacteria in the gut consume H_2_, the surplus produced can still make its way to the bloodstream. An increase in H_2_ production may surpass the rate of augmentation in the H_2_-consuming bacterial population, enabling sufficient H_2_ formation within the body. Experimental evidence now substantiates that the ingestion of H_2_-producing foods can enhance internal H_2_ levels [85,87,93]. Therefore, despite the presence of H_2_-consuming microbial communities in the gut, a robust supply of H_2_ may still retain potential therapeutic efficacy against eye diseases, assuming sufficient systemic H_2_ availability is maintained. If further research can delve into the exact relationship between gut microbiota and H_2_ production, it may offer new insights for optimizing the H_2_ therapeutic strategies.

## 5. Molecular Hydrogen-Mediated Therapeutic Effects on Ocular Diseases

Light permeates through every layer of the eye, leading to the generation of a significant volume of ROS. External factors that harm the eye, such as prolonged hyperglycemia and increased intraocular pressures, expedite the process of oxidative stress. Consequently, research focused on ROS has expanded in various ocular pathologies, encompassing conditions like glaucoma, cataract, AMD and retinopathies. It is hypothesized that the development of these diseases occurs when there is an imbalance between ROS generation and the body’s capacity to neutralize them with natural defense mechanisms, including the production of endogenous antioxidants. H_2_ is very suitable for preventing or improving ocular diseases due to its unique properties. Compared to most known antioxidants, H_2_ possesses advantageous distribution characteristics. It has the ability to permeate biomembranes and diffuse throughout key cellular compartments such as the cytosol, mitochondria, and nucleus. Then, H_2_ is able to specifically eliminate the cytotoxic free radical (•OH and ONOO^−^), as a typical redox reaction. H_2_ can also increase the expression of Nrf2 mechanisms and endogenous antioxidant enzymes, including NRF2, SOD, PGC-1α, glutathione peroxidase, glutathione reductase, glutathione S-transferase and so on. Additionally, H_2_ therapy can also reduce the expressions of stress transcription factors such as NF-kB, thereby exerting leads anti-inflammatory and anti-apoptotic effects on ocular cells.

### 5.1. Molecular Hydrogen-Mediated Therapeutic Effects on Dry Eye Disease (DED)

DED is a common ocular surface disease that can resulting in inflammation, foreign body sensation and blurred vision in patients [94]. DED can be caused by the reduced production of tears, the excessive evaporation of tears, or a combination of these two processes [95]. Accumulating evidence suggest that ultraviolet radiation, pollutants (particular PM 2.5, gaseous, ozone), aging and microbial antigens all lead to increased ROS in the tear fluid film. These unopposed ROS can directly damage the structures such as the tear lipid layer and the myelin sheath of the ocular surface nerve. ROS also cause cellular stress, leading to ocular surface epithelial and goblet cell dysfunction, inflammation and vascular endotheliopathy, ultimately resulting in tear instability [96]. In an animal experiment, researchers established a scopolamine-induced DED model using Wistar rats. The HRS is administered through intraperitoneal (IP) injection in the DED model. The results show that HRS is effective in the treatment of DED [36]. Typically, when indigestible components reach the large intestine, intestinal bacteria utilize them to produce H_2_, together with short-chain fatty acids, carbon dioxide and other substances. H_2_ is absorbed through the small intestinal mucosa and enters the bloodstream through the capillaries in the small intestinal villi. Once absorbed into the bloodstream, H_2_ is transported via the circulatory system to various cells and tissues throughout the body, including the eyeball [97,98]. Via this mechanism, Kawashima, M., et al. developed a novel method to supply large amounts of H_2_ to the human body through intestinal digestion. They added galacto-oligosaccharides (2 g), maltol (2 g) and glucomannan (0.2 g) to a milk solution to generate H_2_-producing milk. H_2_ can be produced in the colon of patients after they have drunk H_2_-producing milk [81]. The results show that the mean value of fTBUT in these DED patients is slowed after drinking. The area under the curve (AUC) of breath H_2_ is approximately 5-fold higher for H_2_-producing milk than for HRW [85].

### 5.2. Molecular Hydrogen Protects the Cornea from Alkali Burn

Alkali burns can cause acute corneal inflammation and corneal neovascularization (NV), both of which are the critical cause of blindness. After alkali burns, the level of ROS in the cornea tissue increases rapidly and subsequently activates the nuclear factor kappa B (NF-κB) [99]. NF-κB plays a significant role in regulating the expression of genes associated with cell growth, inflammation and apoptosis [100]. It translocates into the cell nucleus and promotes the expression of inflammatory cytokines, including vascular endothelial-derived growth factor (VEGF), monocyte chemoattractant protein-1 (MCP-1), IL and tumor necrosis factor alpha (TNF-α) [101]. These cytokines not only promote growth of NV but also recruit more inflammatory cells, which would exacerbating inflammatory response [37]. An experiment studies the H_2_-induced effects on a mouse model with corneal alkali burns. They find that the irrigation of the cornea with H_2_ solution exerts anti-angiogenic effects on the alkali-burned region by inhibiting the mRNA level of NF-κB [37,38].

H_2_ exerts these beneficial effects not only through direct reactions with free radicals but also through indirect pathways involving various anti-oxidative factors. Proliferator-activated receptor gamma coactivator 1α (PGC-1α) is a transcription coactivator that has been shown to mitigate the production of ROS [77]. By binding with nuclear receptor transcription factors and similar elements, it modulates the expression levels of target genes, thereby regulating cellular energy metabolism [102,103]. Functioning as an antioxidant enzyme, the SOD1 protein transforms a pair of superoxide anions byproducts stemming from cellular respiration into oxygen and H_2_O_2_. SOD1 protein plays a crucial role in activating a significant portion of SOD activity, making it an essential element in the body’s defense against oxidative stress [104]. Experimental results show that instilling HRS eyedrops onto the ocular surface of normal rats can increase the activity of SOD1 and PGC-1α in the corneal epithelial tissue. Furthermore, the expression level of VEGF mRNA in the corneal tissue decreases after H_2_ treatment, confirming the H_2_-mediated anti-angiogenic effects on the cornea. Even after ceasing H_2_ administration for over 6 h, these beneficial activities could still be detected in the cornea tissue [38].

### 5.3. Molecular Hydrogen-Mediated Therapeutic Effects on Ultraviolet B Ray-Induced Corneal Damage

Ultraviolet (UV) radiation does not affect our visual perception. However, it primarily causes damage to corneal structures. Compared to visible light, UV carries higher energy, and can induce a range of effects and interactions. For instance, it can trigger chemical reactions and more readily ionize atoms and molecules. Additionally, it has the capability of damaging DNA molecules, posing harm to living organisms. Although the cornea efficiently absorbs more than 90% of ultraviolet B (UVB) radiation, excessive exposure to UVB can lead to serious eye problems, including photokeratitis, corneal epithelial apoptosis and corneal l edema [105,106].

A critical mechanism underlying UVB-induced corneal damage can be ascribed to the over-production of ROS. Under physiological condition, xanthine oxidase activity is distributed in the corneal epithelium, corneal endothelium and lens epithelium. After excessive exposure to UVB, xanthine oxidase activity increases significantly and a notable decrease in xanthine dehydrogenase activity is seen. The primary function of xanthine dehydrogenase enzyme is not the production of O_2_^•−^ and H_2_O_2_, but rather the conversion of xanthine into uric acid, which serves as an effective antioxidant. Xanthine oxidase functions as a xanthine oxidoreductase, generating ROS and exacerbating oxidative stress on the cornea. The reduction in antioxidants, coupled with an increase in ROS, further intensifies corneal damage [107]. Moreover, excessive UVB exposure can result in the reduction in antioxidant level in the cornea. During UVB irradiation, antioxidant enzymes in the corneal epithelium, including SOD, glutathione peroxidase and CAT, are down-regulated. Consequently, a larger burst of ROS will not be counteracted in a timely manner by the antioxidant enzymes and thus accumulate in the cornea [108]. To this end, the exogenous supplement of antioxidants is crucial for protecting the cornea from oxidative damage. A recent study shows that the irrigation of the cornea with H_2_ solution can effectively suppress corneal oxidative stress caused by UVB irradiation in SOD-1-deficient mice or wild-type mice, thereby reducing corneal inflammation and promoting beneficial corneal healing [37].

### 5.4. The Potential Therapeutic Effects on Corneal Endothelial Dysfunction

Endothelial cells play a crucial role in maintaining the transparency of cornea by supporting the pump function [109]. The human corneal endothelium lacks regeneration ability and leaves no opportunity for endogenous supplementation. Corneal endothelial dysfunction can result in stromal edema and the loss of transparency, thereby inducing severe visual impairments in patients [110]. Several etiologies, including Fuchs’ endothelial corneal dystrophy, posterior polymorphous corneal dystrophy and phacoemulsification surgery, can cause endothelial cell dysfunction [111]. For instance, clinical studies have shown that a decreased corneal endothelial density and an increase in cell variability may occur after phacoemulsification surgery [112]. During the phacoemulsification procedure, the probe oscillates at ultrasonic frequencies, creating intense ultrasonic waves that travel through the liquid medium. In this context, small cavitation bubbles are formed, which subsequently enlarge and implode. The heat generated by the implosion of these bubbles leads to the decomposition of water molecules and the production of highly active free radicals (H_2_O→•OH + •H). These toxic ROS can lead to corneal oxidative damage in patients [113,114]. Although corneal transplant is considered the definitive treatment for corneal endothelial dysfunction, there is a global shortage of donated corneal grafts. This dilemma has prompted researchers to explore alternative therapeutic options [115]. Excitingly, a clinical study has shown that the H_2_ dissolved in the flushing solution can reduce corneal endothelial damage during phacoemulsification [116]. Another animal experiment also shows that H_2_ dissolved in the ocular irrigating solution is protective against the against the endothelial cell damage caused by phacoemulsification in rabbit eyes [40]. Researchers have successfully established a corneal endothelium decompensation rabbit model [39]. They find that irrigating rabbit eyes with HRS can improve the survival and physiological function of corneal endothelial cells by inhibiting apoptotic cascades. Furthermore, the H_2_ can mitigate the oxidative stress in corneal tissue via the NF-κB/NLRP3 pathway and the FOXO3A/P53/P21 pathway.

### 5.5. Molecular Hydrogen-Mediated Therapeutic Effects on Cataract

Cataracts are caused by the gradual aggregation or misfolding of crystallin proteins within the lens [117]. Currently, 94 million people worldwide are blind or visually impaired, with cataracts (the clouding of the lens) being the most common cause [118]. The conformational changes of lens proteins are mainly caused by oxidative stress, changes in osmotic pressure and phase separation of proteins and water [119]. Notably, the initial mechanism of cataracts should be ascribed to the lipid peroxidation caused by free radicals (especially •OH) [120]. Lipid peroxidation will affect the permeability of cell membrane, change the internal composition and configuration of lens epithelial cells, lead to malfunction of lens protein and finally cause the occurrence of cataract [19]. H_2_O_2_ is a type of ROS that exists in the aqueous humor of mammals. It can activate a series of signaling transduction events, such as the BCL-2 family, the caspases, NF-κB pathways and the mitogen-activated protein kinases. These signaling will induce the apoptosis of the lens epithelial cell and cause lens opacity [121]. Yang, C. X., et al. injected sodium selenite (25 umol/kg bodyweight) subcutaneously into neonatal Sprague Dawley rats to induce cataracts. Subsequently, they injected HRS (5 mL/kg bodyweight) intraperitoneally every day from 8 to 17 days after the birth of the rats. The severity and development rate of cataracts in the HRS-treated rats was alleviated compared with untreated controls. After an IP injection of HRS, the activity of SOD, CAT, glutathione peroxidase, glutathione reductase and glutathione S-transferase was significantly higher than that of untreated mice [41]. Taken together, these findings suggest that HRS has a salvage effect on the development of cataracts.

### 5.6. The Potential Therapeutic Effects against Uveitis

Uveitis is a medical nomenclature used to describe a collection of diseases characterized by the inflammation of the uvea, which encompasses the iris, ciliary body, and choroid. As a type of intraocular inflammation, the etiology of uveitis is multifaceted, and the pathogenic mechanism is yet unclear [122]. Emerging evidence suggest that ROS plays a critical role in the inflammatory reaction of uveitis. The increased ROS production exacerbates inflammation by promoting the release of pro-inflammatory cytokines and chemokines. Additionally, these cytokines and chemokines contribute to the infiltration of polymorphonuclear leukocytes and macrophages, which further activate the inflammatory cascade [123]. Researchers find that H_2_, may act as a promising medication to treat uveitis. For example, continuous H_2_ inhalation can suppress the release of inflammatory cytokine (MCP-1 and IL-6), thereby alleviating the microglia activation in a rat model of endotoxins-induced uveitis. Moreover, continuous H_2_ inhalation can help to maintain the integrity of the blood–aqueous barrier by reducing the level of prostaglandin E_2_, which mediates the exudation of protein into the aqueous humor [42]. Another study using the same uveitis model also shows that IP injection of HRS is beneficial for the normal function of the blood–aqueous barrier [43]. Therefore, H_2_ can help to mitigate the ROS production in uvea tissue and alleviate the symptoms in uveitis patients.

### 5.7. Molecular Hydrogen-Mediated Therapeutic Effects on Retinitis Pigmentosa

RP is a degenerative retinopathy with genetically heterogeneous features. Photoreceptor degeneration in the RP patients moves from the mid-periphery of the fundus and then progresses toward the macular and foveal regions, with typical symptoms of night blindness emerge first, followed by reduced visual field and, eventually, legal blindness occurs in these RP patients [124]. In RP pathogenesis, the mechanism of rod cell death varies depending on the mutated gene. The initial Rod cell loss would mitigate the oxygen consumption, resulting in elevated oxygen levels in the retinal tissue. Excess oxygen stimulates the production of O_2_^•−^ through mispairing of the electron transport chain in mitochondria and stimulating the NADPH oxidase activity in the cytoplasm. High levels of free O_2_^•−^ can overwhelm antioxidant defenses and produce more reactive species, like ONOO^−^, which is extremely difficult to detoxify. These metabolism alterations will impose constant oxidative stress on the cones. In this context, H_2_ is used to promote cone cell survival and maintain the visual function in RP model [125]. A study has shown that drinking HRW improves the photoreceptor survival and function in rd6 mice [44]. As evidenced by the optical coherence tomography examination, the retinal thickness of the HRW-treated group is significantly higher than that of untreated controls. Histopathological and morphological analysis show that the thickness of outer nuclear layer and the number of opsin red/green (cone opsin) positive cells in HRW-treated mice are higher than those in the control group. RNA sequencing (RNA-seq) analysis shows that there are significant differences in the expression levels of 1996 genes between the two groups. In greater detail, gene and pathway ontology analysis show that the genes responsible for phototransduction are significantly up-regulated in HRW-treated mice. Collectively, these results show that drinking HRW (1.2–1.6 ppm) exerts neuroprotective effect on the cone cell death in RP mice [44]. In another study, researchers deliver HRS into a chemical-induced RP rat model through IP and IV injections. The results show that the average cone density of HRS treatment rats is significantly larger than that of untreated controls, IV injection group has a better therapeutic effect than the IP injection group. Mechanism study shows that HRS treatment increases the level of retinal SOD and reduces the level of retinal MDA in the RP rat model. HRS treatment can modulate the expression level of severe apoptosis-associated genes, such as the BAX, BCL-2, Calpain-2, and caspase-3s [45].

### 5.8. Molecular Hydrogen-Mediated Therapeutic Effects on Diabetic Retinopathy

DR is a progressive complication of diabetes with complex metabolic and pathological features. It is characterized by the microvascular damage and visual impairments. It stems from the chronic and progressive effects of diabetes, causing a sequence of retinal microvascular leaks and obstructions. This cascade results in an array of retinal abnormalities, including microaneurysms, hard exudates, cotton-wool spots, neovascularization, vitreous proliferation, macular edema and, potentially, retinal detachment. The excessive production of ROS can induce structural and functional abnormalities in the retinal microvasculature, leading to the breakdown of the BRB and promoting the progression of DR [126,127]. Additionally, ROS can upregulate the expression level of VEGF and enhance the sessility of VEGF receptors. The overactivation of VEGF leads to the growth of the choroid neovascular, which are also known as choroidal neovascularization (CNV) [128,129].

Compared to other tissues, the retina has the highest oxygen consumption and glucose oxidation levels, making it extremely susceptible to the impacts of oxidative stress [130]. In diabetic patients, there is a decrease in the capacity of antioxidant enzymes to clear free radicals and maintain the redox homeostasis in the retina [131]. For instance, the levels of intracellular antioxidant glutathione are significantly reduced in the diabetic retina [132]. On the other hand, studies have found elevated levels of O_2_^•−^ and H_2_O_2_ in the retinas of a DR rat model and retinal cell lines cultured in high-glucose media [133,134]. Studies indicate that oxidative stress not only propels the progression of DR but also poses a challenge for its reversal due to the buildup of damaged molecules and ROS, even under well-maintained blood glucose levels [135]. In pioneering studies, HRS has been used of treat the streptozotocin (STZ)-induced DR rats. HRS treatment can inhibit the apoptosis caspase in retinal cells, reduce vascular permeability and alleviate the retinal thinning in the DR rat model [46]. This study evaluates HRS-induced effects on the neurovascular and oxidative stress in DR rats. HRS treatment mitigated diminished b-wave amplitudes and oscillatory potentials, BRB breakdown and histological changes in the inner retina in STZ-diabetic rats. HRS ameliorates the oxidative stress and enhance the antioxidant enzyme activity in the DR rats [46]. Additionally, another study uses HRS to treat the oxygen-induced retinopathy in mice. Researchers find that HRS treatment alleviates the retinal neovascularization by inhibiting the mRNA and protein levels of VEGF expressions [47].

### 5.9. Therapeutic Potential of Molecular Hydrogen in Glaucoma

Glaucoma is a group of neurodegenerative diseases that is characterized by optic nerve head damage, the progressive death of RGCs (retinal ganglion cells) and visual field defects [136,137]. Despite the heterogeneous backgrounds of glaucoma, these phenotypes eventually cause the pathological outcome of RGC demise [138,139]. Accumulated evidence obtained from both clinical and experimental research convincingly suggests that oxidative stress is implicated in the RGC loss of glaucoma [140,141]. Oxidative stress can impair the structure of trabecular meshwork, leading to the obstruction of aqueous humor. The intraocular pressure increases significantly, which subsequent results in the retinal ischemia/reperfusion (I/R) injury and RGCs [142,143]. Oxidative stimulus can also attack directly the RGCs degeneration. For example, ROS has been shown to trigger RGC-5 cells (an immortalized RGC line) apoptosis in vitro through the caspase-independent pathways [144]. H_2_ can slow down the development of glaucoma and rescue RGCs. In a rat retinal I/R model, researchers find that the IP injection of HRS can alleviate RGCs apoptosis through the mitigation of DNA oxidation and the overactivation of poly (ADP-ribose) polymerase-1, a nuclear enzyme that promotes caspase-mediated cell apoptosis [48]. In another therapeutic experiment, it has been found that inhaling H_2_ for 1 h daily for 7 consecutive days can significantly lessen RGC loss in a rat retinal I/R model [48]. A mechanism study shows that H_2_ significantly reduces the levels of IL1-β, TNF-α and 4-HNE in the retinal tissue [49]. These findings highlight that H_2_ may be developed into an efficient clinical treatment for glaucoma.

### 5.10. Therapeutic Potential of Molecular Hydrogen in Age-Related Macular Degeneration

AMD is a degenerative retinopathy that causes severe visual impairments in the elder population [145]. Oxidative stress-induced RPE cell dysfunction plays a crucial role in the pathogenesis of AMD [146]. Oxidative stress can lead to mtDNA damage and mitochondrion malfunction in RPE cells. These impairments accumulate with age in the macular region [147]. RPE cells play an important role in regulating the retinal function, including nutrient supply, maintaining orientation and clearing metabolic products [148]. Therefore, RPE degradation can cause photoreceptor death and the loss of central vision [149]. Clinical studies have shown that dietary supplements of antioxidants can effectively improve the vision quality of AMD patients [150]. In this context, it is conceivable that exogenous H_2_ supplementation can serve as a measure to safeguard the retina functioning and alleviate oxidative stress in AMD patients. Researchers have shown that intragastrical administration of HRS can alleviate the oxidative stress in an NaIO_3_-induced AMD mice model by enhancing the aging-related protein (SIRTUIN 3) expression and inhibiting cell senescence [50]. Moreover, they found that HRS can protect the retina tissue by reducing DNA alkylation and promoting DNA repair. Another experiment also provides a more comprehensive description of the therapeutic effect of HRW on an NaIO_3_-induced AMD mice model [51]. It demonstrated that the intragastric administration of HRW can reduce the deposition of yellow-white drusen-like structures and improve the rate of photoreceptor survival and the integrity of retinal vessels. These beneficial effects should be attributed to the reduced MDA content and upregulated SOD level. H_2_ can also alleviate the photoreceptor apoptosis by inhibiting the expressions of caspase 8 and caspase 9. Some researchers use a laser-induced CNV mouse model to simulate the neovascular AMD [52]. The results show that H_2_ inhalation can inhibit the inflammation of an injured retina after Bruch’s membrane disruption, suppress CNV development and decrease the leakage of the CNV. The potential mechanism of action may involve the suppression of the VEGF-dependent pathway of CNV formation through the downregulation of VEGF and HIF-1α. Additionally, it may inhibit the expression of pro-inflammatory mediators, such as TNF-α and IL-6. This mechanism could help reduce the pathological angiogenesis associated with CNV and mitigate the inflammatory response in the retina tissues.

Excessive exposure to blue light is a risk factor of AMD. Blue light between 450 and 500 nanometers can adjust biological rhythms, while the short-wave blue light within 400 to 450 nm is truly harmful. Short wave blue light has high energy, which enables them to penetrate the anterior segment and directly contact the retina. Typically, excessive exposure of blue light can lead to photochemical damage in the retina. Blue light itself can cause a significant increase in ROS production, which will lead to lipid peroxidation and photoreceptor apoptosis [151]. According to previous reports, blue light induces high levels of H_2_O_2_ and O_2_^•−^ and disrupts the mitochondrial respiratory chain, leading to intracellular oxidative stress [152,153]. Notably, blue light at 415–455 nm is the spectral band that most conducive to produce larger bursts of ROS [151]. These ROS can activate the endoplasmic reticulum stress-C/EBP homologous protein (ER stress-CHOP) apoptosis signaling in RPE [154]. The RPE loss can directly lead to the dysfunction and degradation of photoreceptors, thereby accelerating the development of AMD. Excitingly, H_2_ can alleviate the blue light-induced retinal damage. Researchers use blue light irradiation for 6 h to induce retinal damage in rats [53]. The experimental results show that the retinas in HRS-treated rats are less damaged compared with those in the untreated controls. Another independent study shows that the amplitude of electroretinogram waveform and the average thickness of outer nuclear layer in rats injected intraperitoneally with HRS are significantly higher than those in the untreated controls, indicating that HRS protects photoreceptors from light-induced damage [53].

### 5.11. Therapeutic Potential of Molecular Hydrogen in Optic Nerve Crush

As a neuroprotective agent, H_2_ can prevent oxidative stress and inflammation in many neurological diseases. In ophthalmic practice, H_2_ can afford some beneficial effects in treating optic neuropathy. Traumatic optic nerve injury is a serious complication of traumatic brain injury, where the mechanical pressure crushes the optic nerve and causes axonal degeneration. This damage eventually causes neural degeneration, leading to RGC loss and complete blindness in patients [155,156]. Nevertheless, oxidative stress is now considered as one of the most important factors that mediate the above process. In addition, glutamate-induced retinal excitotoxicity acts as another pathogenic mechanism of the neuronal damage in optic nerve crush (ONC). A study shows that the IP injection of HRS for consecutive 14 days can promote the survival of recurrent ganglia cells in a ONC rat model [55]. Researcher have also shown that IP or IV of HRS in rats can promote glutamate clearance and enhance the survival of RGCs [56]. During the early stages of neuronal damage, microglia are activated by glutamate to release inflammatory mediators and toxic substances in the retina [157]. Studies report that HRS can inhibit the microglia the activation in the glutamate-induced retinal toxic injury model in white guinea pigs [56]. NO is an essential signaling factor in oxidative-mediated neuron damage. Another study shows that introducing H_2_ gas into a retina incubator can alleviate the cell loss in the GCL and the INL through neutralizing the ONOO^−^ [57]. Accordingly, the H_2_-mediated effects on oxidative stress or glutamate toxicity may support the hypothesis that H_2_ can protect the optic nerve in ONC through the aforementioned effects.

## 6. Conclusions and Prospects

As a powerful antioxidant, the H_2_-mediated benefits are mainly based on its antioxidant, anti-inflammatory and anti-apoptotic properties. It exhibited remarkable potential for the treatment of diseases of the nervous system (such as stroke, Parkinson’s disease), cardiovascular diseases (such as I/R injury, hypertension) and liver diseases (such as fatty liver, liver fibrosis) [158,159,160,161,162,163]. Between 2016 and 2023, H_2_ therapy gained substantial recognition as a treatment approach for ophthalmic diseases. Notably, researchers have forayed into novel territory by employing H_2_ in animal models to address a broad spectrum of ophthalmic conditions, including the corneal endothelial injury, uveitis and AMD, among others. Furthermore, H_2_ has formally entered the realm of clinical research for ophthalmic diseases, encompassing conditions like cataracts and dry eye disease. This progression marks a significant stride in harnessing the potential of H_2_ in ophthalmologic practice. In clinical practice, the bag containing the ocular irrigation solution is placed in an acrylic vacuum chamber (SNS-type, Sanplatec Corporation, Osaka, Japan) for a period of 24 h. Throughout this treatment, the chamber is purged of air and replaced with 100% H_2_ gas. Subsequent analysis suggests that the concentration of dissolved H_2_ within the solution has successfully achieved a level of 61.9%. During phacoemulsification surgery for cataract patients, this solution containing dissolved H_2_ has been used for irrigation. The endothelial cell density reduction rate in the H_2_ group is significantly smaller compared with that observed in the control group at 1 day, 1 week and 3 weeks postoperatively. Additionally, the results of slit-lamp photography and specular microscopy show that the corneal edema is apparently milder in eyes treated with H_2_ solution post operation [116]. Currently, H_2_ drinks for clinical use are also available. They are packaged as HRW in 420 mL plastic aluminum packs equipped with a gas-tight cap (Aquastamina-R, Nutristamina, Ostrava, Czech Republic). As per the manufacturer’s specifications, HRW is generated from drinking water that has undergone chlorine removal and the subsequent infusion of H_2_ directly into the water under high pressure. This manufacturing process ensures the creation of a H_2_-rich beverage with specific packaging to maintain its integrity and therapeutic potential for clinical applications. It has been clinically used for the treatment of glaucoma. However, the rapid intake of 1260 mL of both HRW and H_2_-free water causes a statistically significant increase in IOP compared to the baseline in healthy individuals. It can be explained that just drinking water could cause parasympathetic nervous system stimulation, which may disrupt the functionality of Schlemm’s canal, ultimately resulting in an elevation of intraocular pressure [164].Current limitations in usage method, including special requirements for H_2_ preparation and storage, as well as inconveniences and potential explosion risks, may hinder the widespread application of H_2_. It is difficult to realize the stable and sustained release of H_2_ through ingesting HRW/HRS. The optimal dosage, injection approach and treatment duration for HRW/HRS have not been clearly defined, demanding the further optimization of therapeutic plans. However, H_2_ production by intestinal bacteria has emerged as a novel method for H_2_ treatment. Thus far, the H_2_-producing milk has been used to treat DED in clinical experiment. Some large-scale clinical studies are necessary to validate its effectiveness and safety during application. At present, research has demonstrated the ability of H_2_ to induce tumor cell damage and apoptosis [165]. The therapeutic effect of H_2_ gas in ocular tumors may be a fruitful area for further work. As a therapeutic drug that is almost flawless, the therapeutic effect of H_2_ on ophthalmic diseases is undisputed. Considerably more prospective clinical studies with larger sample sizes are needed to ensure that standardized H_2_ medication goes to the market.

## Figures and Tables

**Table 1:** Experimental studies on the ophthalmological application of H_2_.

Ocular Diseases	Experimental Objects	Disease Models	Methods of H_2_ Uptake	Effect of H_2_	Refs.
Dry eye disease	Wistar rats	Scopolamine-induced DED	Intraperitoneal injection of HRS (0.6 mmol/L) at a dose of 5 mL/kg BW daily for 28 days; dropping HRS into the eye 1 time per hour, 9 times per day	Inhibiting the activity of NF-kB to reduce inflammation	[36]
Alkali burn of cornea	SOD-1−/− mice or WT mice	Alkali burn	Irrigated with HRW (0.5–0.6 ppm) onto the cornea for 30 min	Reducing oxidative stress; inhibiting angiogenesis in cornea	[37]
	Wistar rats		Irrigated with HRW (1.2–1.6 ppm) onto the cornea for 5 min	Upregulating the expression of antioxidants	[38]
UVB	SOD-1−/− mice or WT mice	Ultraviolet B ray-induced corneal damage model	Irrigated with HRW (0.5–0.6 ppm) onto the cornea	Reducing oxidative stress; inhibiting of angiogenesis in cornea	[37]
Corneal endothelial dysfunction	Rabbits	MNU-induced corneal endothelial cell injury	Irrigated with HRS (1.2 ppm) 3 times a day for 3 min and 3 drops per second for 7 days	Anti-apoptotic effect through the NF-κB/NLRP3 and FOXO3a/p53/p21 pathway	[39]
	Rabbits	Corneal endothelial dysfunction induced by phacoemulsification	Ultrasound oscillation with irrigation solution at almost 61% H_2_ dissolved concentration for 30 s	Reducing oxidative stress	[40]
Cataract	Rats	Selenite-induced cataract	Intraperitoneal injection of HRS (0.6 mmol/L) at a dose of 5 mL/kg BW daily from postnatal day 8 to postnatal day 17	Maintaining the activity of antioxidant enzymes, inhibiting lipid peroxidation	[41]
Uveitis	Rats	Endotoxin-induced uveitis	Inhaling mixed gas that consisted of 67% H_2_ and 33% O_2_ for once a day for 3 weeks	Suppressing the microglia activation	[42]
	Rats		Intraperitoneal injection of HRS (0.6 mM) at a dose of 10 mL/kg BW once a day for 1 week	Maintaining the integrity of the blood–aqueous barrier	[43]
RP	Rats	Rd6 rats	Drinking HRW (1.2–1.6 ppm) 3.42 ± 0.14 mL/day for 1 week	Neuroprotective effect	[44]
	Rats	MNU-induced RP	Intraperitoneal (10 mL/kg BW) and intravitreous (8 μL) injections of HRS (0.6 mmol/L)	Increasing the level of SOD, modulating the expressions of apoptosis-related genes	[45]
DR	Male rats	Rats with streptozotocin-induced diabetes mellitus	Intraperitoneal injections of HRS (0.86 mmol/L) at a dose of 5 mL/kg BW daily for 1 month	Reducing oxidative stress; preserving synaptophysin and BDNF levels	[46]
	C57BL/6J mice	Rats with diabetes mellitus	Intraperitoneal injections of HRS	Reducing the retinal neovascularization, and the expression of VEGF and MDA	[47]
Glaucoma	Rats	Retinal ischemia/reperfusion	Consecutive peritoneally injected of HRS (0.6 mM) at a dose of 5 mL/kg BW until the rats were sacrificed	Alleviating apoptosis of RGCs by overactivating PARP-1	[48]
			Inhaling mixed gas that consisted of 67% H_2_ and 33% O_2_ for 1 h daily for 7 days	Lessening RGCs loss; reducing the levels of IL1-β, TNF-α and 4-HNE	[49]
AMD	Mice	NaIO_3_-induced AMD	Intragastric administration of HRS (4.0 mg/L) at a dose of 10 mL/kg BW for 12 days.	Inhibiting cellular senescence; maintaining DNA homeostasis	[50]
			Intragastric administration of HRW (0.55~0.65 mM) at a dose of 1 mL/g three times daily for 12 days	Inhibiting oxidative stress and apoptosis	[51]
		Laser-induced CNV mouse	Inhaling mixed gas that consisted of 21% oxygen, 42% H_2_ and 37% nitrogen gas for 2/5 h daily for 15 days	Alleviating CNV leakage	[52]
Light-induced retinal damage	Rats	Blue light-induced damage model	Intraperitoneal injection of saturated HRS (0.6 mmol/L) at a dose of 1 mL/100 g BW once a day before and during the exposure session	Suppressing photo-oxidative stress	[53]
			Intraperitoneal injection of saturated HRS(5 mL/kg) before and within 5 days after light exposure		[54]
Optic nerve injury	Rats	Establishing the optic nerve crush model via surgery	Intraperitoneal injection of saturated HRS (5 mL/kg) at 6:00 and 18:00 lasting for 2 weeks	Reducing the lipid peroxidation and apoptosis of RGCs	[55]
	Guinea pigs	Glutamate-induced retinal injury model	Intravitreous (almost 0.6 mmol/L) and/or peritoneal injection (5 mL/kg) of HRS	Clearing glutamate by increasing EAAT-1; reducing RGCs apoptosis of by upregulating GRP78	[56]
	RGCs cells	S-nitroso-N-acetylpenicillamine-induced oxidative stress model	Culturing cell in medium consisting of 5% O_2_ and 95% H_2_ (*v*/*v*) for 24–72 h	Suppressing ONOO^−^-mediated oxidative stress by clearing peroxynitrit	[57]

Abbreviations: H_2_, molecular hydrogen; DED, dry eye disease; HRS, hydrogen-rich saline; NF-κB, nuclear factor kappa B; SOD-1, superoxide dismutase-1; HRW, hydrogen-rich water; MNU, N-Nitroso-N-methylurea; NLRP3, NOD-like receptor family pyrin domain containing 3; FOXO3a, forkhead box O3; BDNF, brain-derived neurotrophic factor; VEGF, vascular endothelial-derived growth factor; MDA, malondialdehyde; AMD, age-related macular degeneration; CNV, choroidal neovascularization; EAAT-1, excitatory amino acid transporter 1; RGCs, retinal ganglion cells; GRP78, glucose-regulated protein 78; WT, wild-type; BW, bodyweight; ONOO^−^, peroxynitrite.

**Table 2:** Current H_2_ therapy methods.

Method	Advantages	Disadvantages	Preparation Method	Administration Method	Ref.
H_2_ gas	Rapid and reliable	Risk of explosion, requires strict management and monitoring	Prepared using H_2_ generators	Direct inhalation of H_2_ gas under professional guidance	[2,74]
	Insignificant effect on blood pressure	Inconveniences during usage			
HRW/HRS	Simple administration through drinking or injection, easily accepted by patients	Limited storage and duration in the body, limited treatment effect	H_2_ gas injection under high pressure	Oral ingestion or drinking, controlled dosage and frequency as per medical advice	[36,38,56,75,76,77,78,79,81]
	Partly mitigates the risk of direct H_2_ gas usage		Reaction between metals and water	Injection under professional healthcare personnel	
			Electrolysis to produce HRW/HRS	Through drip infusion, controlled drip rate and dosage	
				Local eye drops	
H_2_ produced by intestinal bacteria	Production of H_2_ by intestinal bacteria, providing longer duration of effect	Variability due to individual differences in gut microbiota affecting H_2_ production	Induced by ingesting non-digestible substances	Through normal dietary intake of non-digestible components	[82,84]

Abbreviations: H_2_, molecular hydrogen; HRS, hydrogen-rich saline; HRW, hydrogen-rich water.

## Data Availability

Data sharing is not applicable.
